# Effect of Moderate to Vigorous Physical Activity Intervention on Improving Dementia Family Caregiver Physical Function: A Randomized Controlled Trial

**DOI:** 10.4172/2161-0460.1000253

**Published:** 2016-08-09

**Authors:** Carol J Farran, Caryn D Etkin, Amy Eisenstein, Olimpia Paun, Kumar B Rajan, Cynthia M Castro Sweet, Judith J McCann, Lisa L Barnes, Raj C Shah, Denis A Evans

**Affiliations:** 1 Adult Health and Gerontological Nursing, Rush University Medical Center, 600 South Paulina, AAC Suite 1080, Chicago, IL, 60612, USA; 2 American Joint Replacement Registry, 9400 West Higgins Road, Rosemont, IL, 60018, USA; 3 CJE Senior Life, 3003 W. Touhy Avenue, Chicago, IL 60645, USA; 4 Community, Systems and Mental Health Nursing, Rush University Medical Center, 600 South Paulina, AAC Suite 1080, Chicago, IL, 60612, USA; 5 Department of Internal Medicine, Rush Institute for Healthy Aging, Rush University Medical Center, 1645 West Jackson, Suite 675, Chicago, IL 60612, USA; 6 Department of Medical Affairs, Omada Health, Hoover Pavilion, Room N229, 211 Quarry Rd, Palo Alto, CA 95305-5705, USA; 7 Rush Institute for Healthy Aging and Adult Health and Gerontological Nursing, Rush University Medical Center, USA; 8 Neurological Sciences and Behavioral Sciences, Rush Alzheimer’s Disease Center, Rush University Medical Center, 600 S. Paulina, Chicago, IL, 60612, USA; 9 Department of Family Medicine and Rush Alzheimer’s Disease Center, Rush University Medical Center, 600 S. Paulina, Suite 1022, Chicago, IL, 60612, USA

**Keywords:** Alzheimer’s disease, Family caregiving, CHAMPS, Senior physical fitness tests: 2 min step test and 30 s chair stand test

## Abstract

**Objective:**

Alzheimer’s disease and related dementias (ADRD) affect more than five million Americans and their family caregivers. Caregiving creates challenges, may contribute to decreased caregiver health and is associated with $9.7 billion of caregiver health care costs. The purpose of this 12 month randomized clinical trial (RCT) was to examine if the Enhancing Physical Activity Intervention (EPAI), a moderate to vigorous physical activity (MVPA) treatment group, versus the Caregiver Skill Building Intervention (CSBI) control, would have greater: (1) MVPA adherence; and (2) physical function.

**Methods:**

Caregivers were randomly assigned to EPAI or CSBI (N=211). MVPA was assessed using a self-report measure; and physical function was objectively assessed using two measures. Intention-to-treat analyses used descriptive, categorical and generalized estimating equations (GEE), with an exchangeable working correlation matrix and a log link, to examine main effects and interactions in change of MVPA and physical function over time.

**Results:**

At 12 months, EPAI significantly increased MVPA (*p*=<0.001) and number of steps (*p*=< .01); maintained stable caregiving hours and use of formal services; while CSBI increased hours of caregiving (*p*=<0.001) and used more formal services (*p*=<0.02). Qualitative physical function data indicated that approximately 50% of caregivers had difficulties completing physical function tests.

**Conclusion:**

The EPAI had a stronger 12 month effect on caregiver MVPA and physical function, as well as maintaining stability of caregiving hours and formal service use; while CSBI increased caregiving hours and use of formal services. A study limitation included greater EPAI versus CSBI attrition. Future directions are proposed for dementia family caregiver physical activity research.

## Introduction

Alzheimer’s disease and related dementias (ADRD) currently affect more than five million Americans. Research notes that caring for a person with dementia poses numerous challenges due to continued advancement of the disease and eventually, may contribute to decreased caregiver health. Dementia caregivers, compared to non-caregivers, have reported fair to poor general health, that caregiving has made their health worse and that they became more frail prior to their care recipient’s death [1–3]. The physical and emotional impact of dementia caregiving has been associated with $9.7 billion in health care costs in the United States [2]. Caregiving is a complex process that is affected by the care recipient’s dementia severity, caregiver’s perceptions of care-related challenges and responsibilities, and caregiver’s personality and available resources. These complex factors must be considered when addressing the health impact of caregiving responsibilities and developing interventions designed to protect caregiver health [2].

Over the past 30 years, more than 200 effective psychoeducational randomized controlled trials (RCT) have been conducted with dementia family caregivers [4]. The majority of these interventions, however, have focused on psychoeducational, counseling and psychotherapeutic interventions, as well as skill building, case management, support groups, respite care, training of the person with dementia and other multicomponent approaches [2,5–7]. These interventions have focused on the caregiving process, including understanding dementia, managing behavioral symptoms of dementia, providing personal care, reducing caregiver stress, and finding and using community-based services. Nearly 15 years ago, researchers suggested [8] that more family caregiver interventions should be placed in a public health context, thus positioning these interventions to have a greater impact on promoting caregiver health and wellness.

Physical activity has been identified as one of the best approaches for improving physical and mental health [9]. However, few known family caregiver physical activity interventions have been conducted. The first known 12 month randomized controlled trial (RCT) with dementia family caregivers examined health and quality-of-life effects of moderate-intensity exercise compared to a nutrition intervention in 100 sedentary older female family caregivers [10]. The intervention resulted in improved sleep quality, total energy expenditure and stress-induced blood-pressure reactivity, as well as improved caregiver perceived stress, burden and depression. A total of 74% of treatment-group caregivers adhered to the three weekly exercise sessions for an average of 35 min/ session [11]. A second 12 month multicomponent intervention tested a telephone-based physical activity intervention with 137 female spousal caregivers. Outcomes included increased physical activity and exercise self-efficacy, and decreased perceived stress at 6 and 12 months [12]. Competing caregiver demands and depressive symptoms were barriers to program retention and adherence. A limitation of these studies was that they focused on older White women. More recently, a small international study (N=31) built further upon this work. This RCT reported that treatment group caregivers compared to controls, were able to increase MVPA (≥ 3 Metabolic equivalents [METS]) to 3 times/ week for 12 weeks and also reported significant reductions in caregiver burden, frequency of caregiver fatigue and improvement in sleep quality [13]. A fourth caregiver physical activity RCT, recently completed by our research team, used the same database reported in this manuscript, but focused on secondary mental health outcomes including: subjective burden, depressive symptoms and positive affect. The treatment group significantly increased caregiver total and total moderate to vigorous physical activity (MVPA) and showed greater positive affect at both six (*p*=0.01) and 12 months (*p*=0.03); improved burden at 3 months (*p* 0.03); but had no significant effect on depressive symptoms [14]. Three other family caregiver-related physical activity studies were excluded from this review for noted differences [15]: Hill et al. did not specify care recipient diagnosis [16]; Mardsen et al., focused on stroke survivors [17] and Teri et al., included a physical activity intervention delivered *by* family caregivers *to* persons with dementia [18].

These caregiver physical activity interventions demonstrated that caregivers were able to increase total and total moderate-intensity physical activity; make positive changes in stressors and caregiver resources; and improve some aspects of mental health [10–14]. Implications of these studies suggested that (a) individualized home-based telephone interventions are preferred over groups; and (b) caregivers prefer moderate-intensity programs that are simple, non-competitive and consist of shorter bouts of activity. Assisting older caregivers to increase moderate and vigorous physical activity (MVPA)-known to positively impact physical function, mental and physical health-may offer protective effects for stressed caregivers and enable them to better maintain their caregiving role and their own health for a longer period of time [19].

The purpose of this study was to: (1) Examine the context of dementia family caregiving at Baseline and 12-months concerning caregiver and care recipient socio-demographic characteristics; and caregiver stressors, resources and background health to determine if there were differences between the EPAI and CSBI at baseline and 12 months; and (2) Test the hypotheses that the EPAI, compared to the CSBI, will: H1: Attain higher MVPA adherence (≥ 150 min/week); and H2: Attain greater physical function using two *Senior Fitness Tests* (i.e., 2 min Step Test and 30 s Chair Stand).

## Methods

This study tested the effectiveness of a 12-month multi-component individualized physical activity intervention. This RCT recruited a community-based sample of strained family caregivers of persons with ADRD on an ongoing basis and assigned them to either the EPAI treatment or CSBI control group. Individualized MVPA was defined as any leisure, ongoing, or planned physical activity that was ≥ 150 min/ week (≥ 3 Metabolic Equivalents [METS]) and adapted to caregiver abilities [20]. The EPAI, a behavior-change intervention, combined content on increasing physical activity with attention to caregiving concerns that might impede such an increase [10]. In contrast, the CSBI control was designed to minimize treatment exposure to physical activity by restricting any reference or focus on physical activity and instead, was tailored after usual-care interventions that focused on care-related education and support, thus being unlikely to have an impact on physical activity [21].

### Participants and procedures

Detailed participant recruitment methods were previously described [22]. Participants were 211 caregivers who met eligibility criteria: (a) ≥ 30 years of age, English speaking, caring for a person with dementia and residing at home; (b) providing ≥ 10 h of unpaid care/week; (c) not participating in MVPA ≥ 60 min/week over the past six months; (d) free of medical/functional conditions that would limit MVPA; (e) reported strain with at least one item from the caregiver health effects study measure of strain [23,24] and (f) no prior participation in a physical activity intervention. Caregivers signed an informed consent before baseline assessment and randomization, confirmed willingness to participate in either study condition, and agreed to increase MVPA if assigned to the EPAI.

This Telephone Resources and Assistance for Caregivers (TRAC) study was conducted by Rush College of Nursing, who had the primary study contract, along with the Stanford Prevention Research Center, who provided consultation and oversight of the physical activity intervention. Review boards from both institutions approved the study.

### Study measures

A comprehensive in-person assessment was conducted in caregivers’ homes at baseline, 6 and 12 months; while 3 and 9 month assessments were conducted by telephone. Assessments were completed by two experienced research associates (RAs) who were trained and monitored by the Project Manager (CDE); retraining occurred if necessary for consistency and accuracy. RAs were blind to treatment assignment. After baseline assessment, the Project Manager randomized participants to either the EPAI or CSBI, using a simple random-sequence table of 1’s and 2’s, generated by the study statistician.

### Primary outcome: Moderate to vigorous physical activity (MVPA)

The Community Health Activities Model Program for Seniors (CHAMPS) measure of MVPA, defined as ≥ 150 min of physical activity/week (≥ 3 METS) was the primary study outcome [20]. This 41-item self-report measure assessed both total and total moderate physical activity, including the range of specific physical activities across all levels of intensity or physical exertion, typically performed by older adults over a one-month time frame. Psychometric properties are well-established and the measure is sensitive to physical activity change in older adults [25]. In an earlier pilot study of this intervention [26] we found significant correlations between self-reported MVPA and the objective waist-worn Mini Mitter (r=0.72, *p*= ≤ 0.01) [27]; and MVPA and pedometer steps (r=0.59 to 0.94, *p*= ≤ 0.05) [28].

### Secondary outcome: Physical function

Two objective assessments from the *Senior Fitness Test* were used: the 2 min Step Test and the 30 s Chair Stand Test to assess lower body strength and aerobic endurance. The *2 min Step Test* required that caregivers step and raise each knee to a point midway between their kneecap and top hip bone (about 12–16 inches) and continue stepping for 2 min. The final score comprised the number of steps that the right knee reached the required height within 2 min. This measure is well-validated and has positive correlations with other similar measures (r=0.73–0.74) (Range 0–100) [29].

The *30 s Chair Stand Test* assessed lower-body strength needed for numerous tasks such as climbing stairs, walking, and getting out of a chair, tub or car. Older adult’s ability to complete this test has also been associated with reduced falls. This objective assessment required that caregivers sit in a comfortable straight chair with their arms crossed over their chest and determine how many times in 30 s they completed full stands from a seated position without using their hands or arms to support or push themselves up. This measure has positive correlations with other similar measures (r=0.71–0.78, for women and men, respectively) [29].

### Caregiver strain, stressors and resources

Strained CGs were selected to maximize intervention change [23]. A 3-item measure determined if CGs had strain with: (a) CR’s personal/ instrumental activities of daily living (PADL/IADL (Yes/No); (b) if CGs provided assistance/arranged others to provide care (Yes/No); and (c) if CGs had mental/physical strain in providing this care (1=*no strain to* 3=*a lot of strain*). Eligibility criteria required that CGs had *some to a lot of* strain with ≥ 1 item [24]. To determine comparability between EPAI and CSBI caregiving situations, three care-related stressors and two resource indicators were assessed. Higher levels of caregiving stressors have been associated with higher CG stress [30]. Stressors included CR’s cognitive impairment, care required for personal and instrumental activities of daily living (PADL /IADL), and number of behavioral symptoms of dementia.

The *Mini Mental State Examination* (MMSE) screened for presence of and dementia severity [31] (Cronbach’s alpha with TRAC sample=0.82), (Range 0–30) where higher scores indicated better CR cognitive function. MMSE scores were obtained in one of two ways: (1) from the RADC (Rush Alzheimer’s Disease Center) registry, where previous consents to share data were provided by CR and CG; RADC staff contacted CGs, informed them about the study and asked if they could share the CG/CR names and contact information with TRAC study staff; and (2) MMSE was obtained in-person by TRAC RAs if no prior assessment was available, with the CR providing consent.

To reduce subject burden, Chicago Health and Aging Project (CHAP) epidemiological study measures assessed caregiver stressors and resources [32]. *Personal and Instrumental Activities of Daily Living* (PADL /IADL), 4-items measured tasks provided by caregivers to their CR: (a) personal (e.g., bathing, dressing), (b) instrumental (e.g. shopping, meals), (c) supervision of others (e.g. doctor visits, adult day care) or (d) health care-related tasks (e.g. wound care, blood pressure). This measure determined if care was provided to their CR within the past week (0=no, 1=yes; Range=0–4) (TRAC sample Cronbach’s alpha=0.83) [32].

*Number of Behavioral Symptoms of Dementia*, a 15-item CG assessment of how many behavioral symptoms for which CR needed supervision within the past week (T R AC sample Cronbach’s alpha=0.64) (0=no, 1=yes; Range 0–15) (e.g. wandering, up at night, physically violent, uncooperative, .repetitiveness, depressed, restlessness/agitated, irritable/angry, suspicious, happy/cheerful, warm/affectionate). *Total hours of caregiving/week* included a sum total of hours of care provided in the past week (Range 1–168) [32].

Two caregiver resource assessments, *Total formal services (TFS)* included five items about specific services used in the past three months: (a) adult day care or early intervention programs, (b) respite services, (c) support groups, (d) caregiver educational activities, or (e) assistance from case manager or financial or legal planner (Range 0–5) [32].

*Perceived social support* (PSS) included four items concerning CG’s perceptions about informal support and whether they had: (a) a special person when needed, (b) a person who was a real source of support, (c) a special person who cared about their feelings, and (d) were satisfied with their support in the past month (Response range 0=Strongly Disagree to 4=Strongly Agree, Range 0–16). TRAC sample Cronbach’s alpha=0.83. Supportive relationships have been shown to reduce vulnerability to stress, depression, and physical illness [33].

### Caregiver socio-demographic variables

*Socio-demographic characteristics* included standardized baseline variables of CG and CR information often used in epidemiologic studies [34].

### Interventions

The TRAC study built upon existing family caregiver intervention research and was guided by two theoretical models: the stress process model which guided the family caregiving intervention [35]; along with a social cognitive interaction model of health behavior that guided the physical activity intervention [33]. The stress process model focused on the caregiving context, using three major variables: (a) caregiver background characteristics (i.e., age, gender, race and employment); (b) caregiver primary stressors (i.e., needs associated with care recipient level of dementia impairment, personal/instrumental activities of daily living, behavioral symptoms, and hours of care provided); and (c) caregiver resources (i.e., formal services and perceived social support [30,32].

Multi-component treatment conditions implemented over 12 months included specified content, identical contact schedules, and specific training materials by intervention. Two PhD-prepared interventionists, one for each condition, had preparation in health behavior change and physical activity (EPAI: AE) and geropsychiatric nursing and family caregiving (CSBI: OP). Identical contacts by intervention included (1) baseline 1- to 1½-h in-home orientation, (2) weekly 15–20 min telephone calls (Weeks 2–8), (3) biweekly 15–20 min telephone calls (Months 3–4), and (4) monthly 15–20 min telephone calls (Months 5–12). Both conditions could provide up to 20 intervention contacts totaling 6–7 h. To reach participants for each session, interventionists made up to three calls. If caregivers could not be reached, interventionists proceeded to the next planned telephone call. Both interventions addressed caregiving content but only the EPAI addressed physical activity content.

#### Enhancing physical activity intervention (EPAI): Treatment condition

The EPAI goal was to increase regular MVPA to the level of ≥ 150 min/weekly (≥ 3 METS), and minimize barriers to increasing physical activity by addressing caregiving and other concerns. The EPAI built upon existing physical activity guidelines [36,37] and Bandura’s [33] social cognitive behavioral approaches such as: (a) *Self-Regulation*, including physical activity goal-setting and structuring of outcome expectations; feedback concerning goals, encouragement of self-rewards, and other reinforcement strategies; (b) *Behavioral Rehearsal*, including self-monitoring for adherence disincentives and agreement on a behavioral contract to solidify caregiver’s commitment to the intervention; (c) *Reciprocal Determination*, between the caregiver and environmental influences, removing obstacles to exercise; using environmental prompts including notes or health-related reading materials to reinforce behavior change; (d) *Self-Reflection*, including personal thoughts and beliefs about exercise and their connection to self-efficacy; and (e) *Vicarious Learning*, where the interventionist acted as a role model and assisted caregivers to learn by sharing their own successful approaches to setting goals, problem-solving, overcoming challenges and engaging in more physical activity. Physical activity goals and plans were individually tailored by the caregiver and interventionist based on caregiver personal needs and preferences [26,38]. The caregiver and interventionist considered baseline activity and personal capabilities, preferences, resources and potential barriers. Caregivers received instructions concerning: use of a pedometer, warm-up and cool-down exercises; rating perceived physical activity exertion and gauging intensity, with the goal of reaching MVPA, considered to be a beneficial cardiovascular level [39]. Caregiving topics emphasized obstacles to increasing physical activity (i.e., managing family member care-related needs, balancing caregiving responsibilities, building in self-care and using available resources).

Regular EPAI telephone calls: (a) reviewed caregiver Physical Activity Logs and average pedometer steps; (b) selected mode/type of physical activity using the FITT principle (Frequency, Intensity, Time and Type [39] and (c) identified barriers to increasing activity. The goal was to help caregivers find the combination of activities that fit their needs, using a gradual approach to reaching optimal physical activity (i.e., 30 min of aerobic physical activity most days/week) while assuring comfort and minimizing risks [9]. Caregivers chose the combination of physical activities most suitable to their abilities and set short- and long-term goals to eventually reach ≥ 150 min/week of moderate-intensity aerobic activities (i.e., walking, biking and calisthenics). Once moderate-intensity goals were met, new goals were set, such as increasing intensity or adding non aerobic activities (i.e., stretching, balance or strength building). Strategies relied on teaching fundamental self-management skills: (a) setting long and short-term goals, (b) self-monitoring to gradually increase activities, (c) identifying activity barriers and practical solutions to overcome them, and (d) identifying mechanisms of relapse prevention.

#### Caregiver skill building intervention (CSBI): Control condition

The CSBI was designed to provide information, support and problem-solving for caregivers by combining stress/coping and social/ cognitive approaches; but precluded any content or discussion regarding physical activity. CSBI content focused on: (a) understanding dementia and safety issues, (b) developing skill in providing person-centered care in responding to personal and instrumental activities of daily living and managing dementia behavioral symptoms, (c) managing caregiver stress, and (d) finding and using formal/informal services[40,41].

Similar social cognitive approaches used in the EPAI were adapted in the CSBI and included: (a) *Self-Regulation*, goal-setting and structuring of outcome expectations regarding caregiver concerns where the CSBI interventionist and caregiver discussed setting realistic goals: what worked/ what did not work; and seeking out personal and caregiving self-rewards; (b) *Behavioral Rehearsal*, involved helping caregivers to self-monitor caregiving areas of difficulty and emotional distress and encouraged problem-solving and adherence to suggestions and recommendations that had been discussed between the CSBI interventionist and caregiver; as well as discussing behavioral contracts to solidify the caregiver’s commitment to trying different approaches; (c) *Reciprocal Determination*, between the caregiver and interventionist provided opportunities to determine environmental influences on changes in the care recipient’s behavior, removing obstacles to improving caregiving approaches; and using environmental prompts including self-reminder notes or health reading materials to reinforce behavior change; (d) *Self-Reflection*, included encouraging personal thoughts and beliefs about caring for their relative and their connection to self-efficacy; and (e) *Vicarious Learning*, providing opportunities where the interventionist anonymously shared successful approaches used by other caregivers to setting goals, problem-solving and overcoming challenges [26]. Caregiving and self-care goals and plans were individually tailored for caregiver needs [40,41].

CSBI telephone assessments addressed (a) most difficult concerns, (b) things going well, and (c) setting weekly goals. Support from the interventionist included active listening/empathy, social-cognitive skills and problem-solving related to caregiving.

#### Intervention implementation and fidelity

Treatment fidelity was monitored independently by two PhD-prepared supervisors (CCS and JJM), one for each condition. Interventionists initially received 8–10 h of individualized training for their respective intervention. Dr. Cynthia Castro Sweet, Co-I, provided EPAI interventionist training which included EPAI content and approaches for increasing physical activity. Drs. McCann, Co-I and Dr. Farran, PI, provided CSBI training based on an earlier group-based Caregiver Skill Building Intervention [41,42].

To monitor intervention implementation, each interventionist/ caregiver telephone call was audio recorded and reviewed by their respective fidelity supervisor [43]. Supervisors reviewed and rated each respective audio recording, using a fidelity checklist: (a) was the intervention implemented as intended (i.e., treatment delivery)? (b) did the caregiver receive the intervention as intended (i.e., receipt)? (c) did the caregiver implement the intervention as intended (i.e., enactment)? and (d) was the intervention protocol maintained over time (i.e., drift) [14]. Each supervisor/interventionist team met separately and biweekly in-person or by telephone, to review adherence to study protocol and address any intervention issues/concerns. Issues or changes that needed to be made were referred to the Principal Investigator and Project Manager for discussion and/or revisions.

### Randomization

The Project Manager (CDE) used data management reports to confirm caregiver eligibility. Once inclusion criteria were met and caregivers expressed interest in the study, the baseline interview was scheduled by one of two blinded research assistants. Data were collected using computer-based direct entry, thus minimizing missing data. After baseline data were collected, the Project Manager randomly assigned caregivers on an ongoing basis to either the EPAI or CSBI using a computerized list of numbers (1’s and 2’s) generated by the statistician. This list was balanced with approximately 7–8 persons/ group for practical and administrative reasons. Treatment assignment was concealed from caregivers and care recipients [14].

### Statistical Analyses

Descriptive analyses were conducted using means and standard deviations for continuous health measures; and frequencies and percentages for categorical data. Intention-to-treat analyses with generalized estimating equations (GEE), with an exchangeable working correlation matrix and a log link was used to study the difference in change of population means over the duration of study period [44,45]. GEE models included main effects and interactions for total MVPA, the primary outcome; and used the *2 min Step Test* to test the secondary outcome. The Student’s t test examined continuous variables to determine if there were differences in socio-demographic, stressor, resource and background health behavior variables by treatment condition and study time at baseline and 12 months. Chi-square analyses examined if there were differences in categorical variables such as selected socio-demographic or other groups (i.e., gender, race/ ethnicity, marital status, CG/CR relationship, living arrangements, level of education, by participant flow through the study, and MVPA adherence). The level of significance for hypothesis testing was set at 5%. Analyses were conducted using SAS Software [45].

#### Sample size

A sample size of 190 caregivers was initially determined based on an a priori hypothesis which proposed a two-way comparison between the EPAI and CSBI groups for increasing weekly minutes of total MVPA. For a Type 1 error rate of 0.05 and a one-sided test, we estimated that we would have 80% power to detect an effect size of 0.395. However, during the study we experienced EPAI differential attrition, so in consultation with our Data and Safety Management Board, we recruited 21 additional caregivers for a total N=211 [44].

## Results

### Participant flow

**Figure 1:**
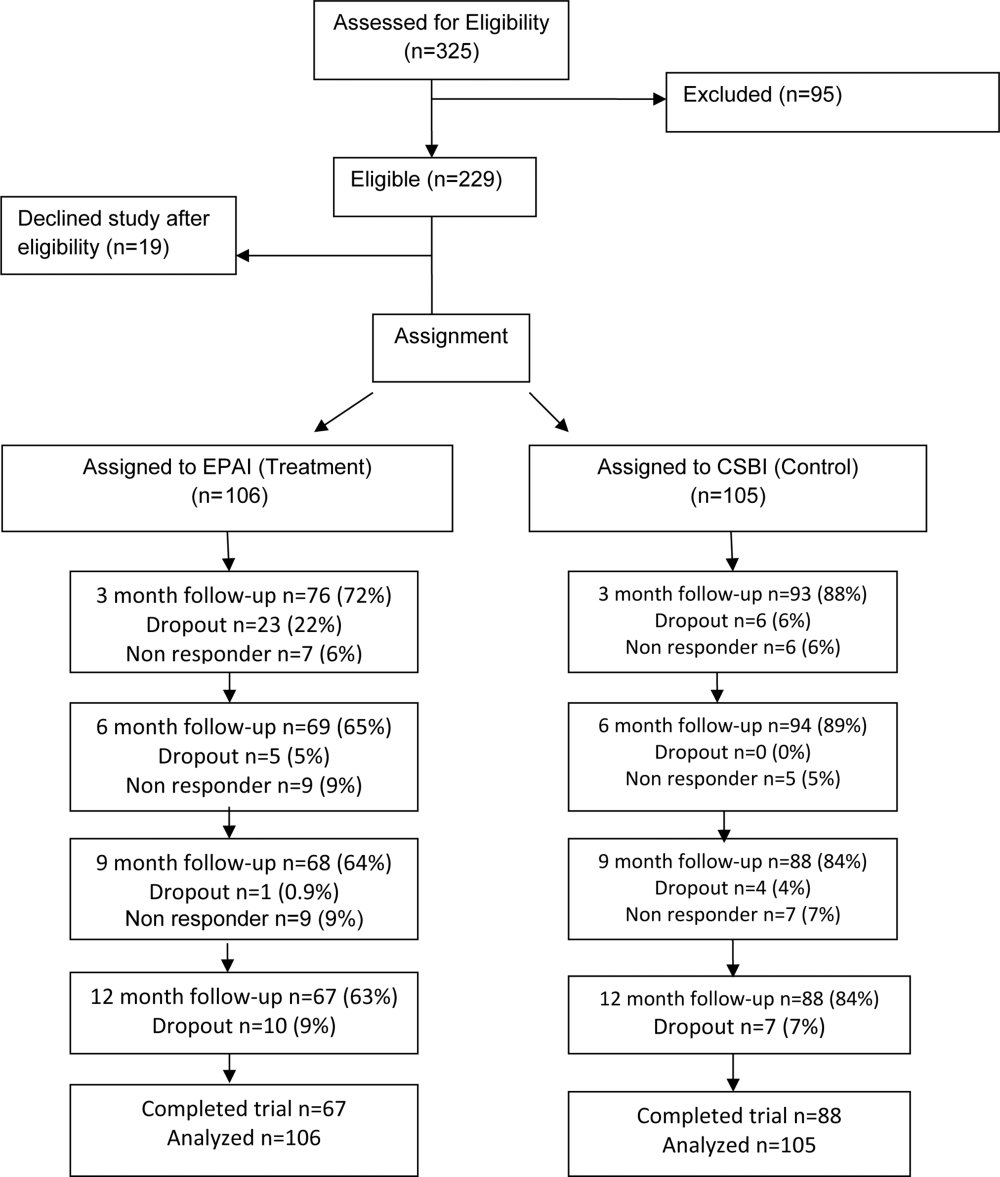
Telephone resources and assistance for caregivers: Study CONSORT table.

The study was conducted from 05/01/07 to 2/28/13, for a total of 70 months. Rolling recruitment occurred for 48 months (01/01/08–12/30/11). Of 211 participants, 73% (*n*=155) completed data collection over 12 months; 63% in the EPAI and 84% in the CSBI (Figure 1) (*p*=0.001 for between-condition differences). The highest percentage of EPAI participants (22%) dropped out by the 3 month follow-up. CSBI dropout occurred more gradually and ranged between 0–7% throughout the study. Early and later EPAI dropouts were identified. Early EPAI dropouts withdrew within the first two intervention weeks, participated in 2–3 intervention sessions and did not complete follow-up assessments (*n*=28).

Early dropouts reported that the EPAI involved more time engaging in physical activity than they expected (74%). Later EPAI dropouts withdrew after participating in approximately 70% of intervention sessions (*M=*14, *SD*=6), they did not complete follow-up interviews, and/or were lost to follow-up (*n*=27). Later dropouts significantly differed from study completers by being younger (*M*=57, SD=14 vs. *M*=62 years, *SD*=11, *p*=0.02) and still employed (*p*=0.05). They expressed demoralization concerning lower levels of MVPA compared to the EPAI (*M*=146, *SD*=175 vs. completers *M*=231, *SD*=377, *p*=0.06). They did not have significantly higher depressive symptoms, burden or lower positive affect (*p*=0.18–0.78); nor did they report higher levels of care-related responsibilities with their relative’s activities of daily living or behavioral symptoms (*p*=0.21–0.71). Their physical health was similar to study completers, where they reported no differences in number of chronic conditions (*p*=0.65), general health (p*=*0.33), systolic or diastolic blood pressure (*p*=0.83–0.85) or BMI (*p*=0.65). The majority of dropouts were White (62%).

### Baseline socio-demographic characteristics

The 211 caregivers enrolled in the study ranged in age from 32–86 years (*M*=61 years, *SD*=12) (*p=0.43*) (Table 1). The majority was female (82%), married (63%), spousal (44%) or an adult child (50%), who lived with their care recipient (89%), and who had more than a high school education (82%). Two-thirds (66%) were non-Hispanic white, 27% were African American and 7% represented other multicultural groups. Slightly over one-third of caregivers were employed (37%). Care recipients (CRs) were approximately 19 years older than caregivers (*M*=80 years, *SD*=10) and most were female (65%). CRs were identified as having Alzheimer’s disease or a related dementia (MMSE, *M=*15.5, *SD=*8) (Table 1). There were no significant baseline socio-demographic differences by intervention group, suggesting that randomization was effective.

### Caregiver stressors, resources and background health

Caregiver stressors, shown in Table 1, included providing care for 2–3 of care recipient’s PADL/IADL (e.g. toileting, bathing) and 7–8 weekly behavioral symptoms (e.g. wandering, up at night, physically violent, uncooperative, repetitive, hiding/hording, depressed or clinging). At baseline, caregivers provided an average of 34 h of care/week, but at 12 months, CSBI hours of care/week significantly increased to an average of 42 h/week (*p*=0.001). Caregivers reported using between 1–2 formal service resources at baseline, although CSBI caregivers slightly increased their formal service use at 12 months and EPAI caregivers slightly decreased their services (*p*=0.02) (Table 1). Caregivers in both interventions reported having between 11–12 persons who provided perceived support for them at baseline and 12 months. Findings concerning caregiver’s background health (Table 1), note a low number of chronic conditions (*M*=2.1, *SD*=1.5) which did not significantly change at 12-months. Caregivers reported taking more medications at baseline (*M*=5.6, *SD*=4.0) than at 12 months (*M=*3.3, *SD=*3.0) which just approached a significant decrease (*p*=0.06). Body mass index (BMI) and weight were high for caregivers in both groups and did not change over 12 months. Blood pressure was within normal limits for both intervention groups at baseline and 12 months.

### Physical Activity

#### Primary hypothesis: Increase moderate to vigorous physical activity (MVPA)

**Figure 2:**
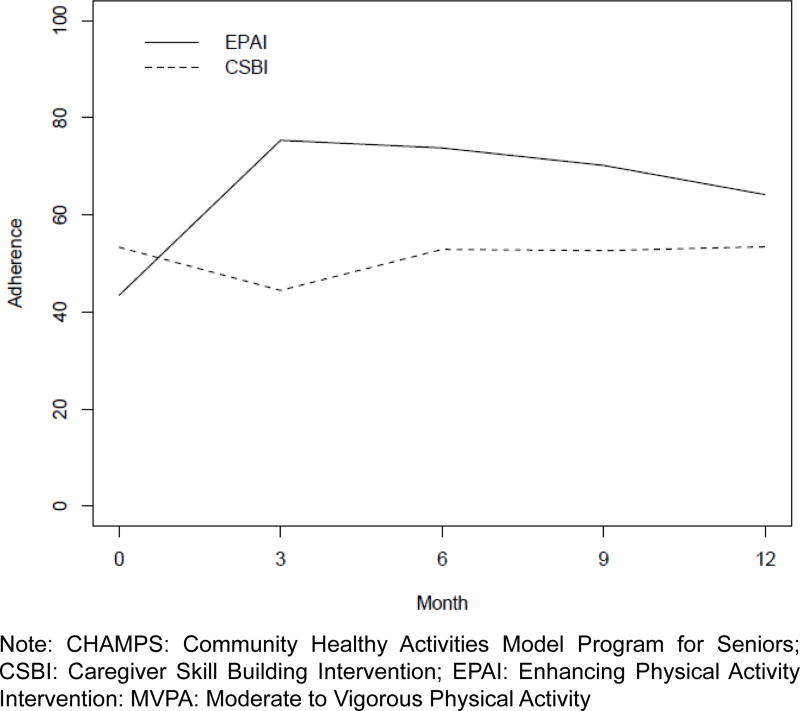
Percentage of Caregiver Adherence to ≥ 150 minutes of CHAMPS MVPA by Week from 3 to 12 Months by Treatment Group

At baseline, EPAI caregivers reported having somewhat fewer MVPA min/week (*M*=62 (*SD*=119) than the CSBI (*M*=79 (*SD*=111) (*p*=0.09). At 12 months the EPAI doubled their MVPA minutes/week while the CSBI decreased their weekly MVPA minutes by 5% (Table 2). The recommended level of MVPA) was for at least 150 min/week. Participants were categorized into: low= ≤ 150 min/week and high= ≥ 150 min/week; where 63% of EPAI and 37% of CSBI adhered to MVPA from 3–12 months (Figure 2). This between-group difference was statistically significant (χ^2^ 1 df=29.37, *p*=<0.0001), thus further supporting Hypothesis 1. Moderate physical activities most frequently reported by caregivers included brisk walking, heavy housework or gardening, or light strength training.

Since baseline and 12 month MVPA was positively skewed for both groups, we log transformed these data for further model testing. To permit full use of longitudinal data, log-transformed generalized linear mixed models were employed using the generalized estimating equation (GEE) approach to evaluate EPAI effects on improving caregiver MVPA by time, main effect and group-by-time interaction (Table 3). For log-transformed MVPA there were significant interactions between the EPAI-by study-month at both 6 and 12 months (*p*=<0.05 and 0.01, respectively), thus providing further support of the primary study hypothesis.

#### Secondary hypothesis: Increase physical function

Two Senior Fitness Test observational physical function assessments were examined at baseline and 12 months: the *2 min Step Test* and the *30 s Chair Stand Test* for assessment of aerobic capacity and endurance (Table 2) [29].

Although means were not significantly different at 12 months for either physical function test, EPAI caregivers showed a 14% increase in the 2 min Step Test, compared to the CSBI decrease of 6% in their steps.

#### Multivariate analyses

To permit full use of longitudinal data, generalized linear mixed models were employed using generalized estimating equations (GEE) to evaluate EPAI effects on improving caregiver physical function of both the *2 min Step Test* and the *30 s Chair Stand* by time, main effect and group-by-time interaction (Table 3). For the *2 min Step Test* there were significant interactions between the EPAI-by study-month at both 6 and 12 months (*p*=<0.05 and 0.01, respectively) suggesting that the EPAI was effective in increasing caregiver number of steps by approximately 10 steps at 6 months (*p*=<0.05) and approximately 13 steps at 12 months (*p*=<0.01) (Table 3). There were no significant group effects for the *30 s Chair Stand* (Data not shown). These data suggested that the *2 min Step Test* supported Hypothesis 2, while data concerning the *30 s Chair Stand* did not [29].

#### Qualitative analysis of 2 min Step Test

Open-ended qualitative comments regarding the Step Test indicated that nearly half of the caregivers (47%) had some difficulties in completing this test. Approximately one-quarter of the caregivers (24%) had high blood pressure (≥ 140/90) or other cardiovascular limitations that interfered with completing this test (i.e., caregiver had recent heart surgery and was still in cardiac rehabilitation; or waiting for cardiologist consult regarding aortic valve replacement). Another 23% of caregivers had functional limitations that interfered with completing this test (i.e., balance difficulties, indicating the need to hold onto the wall or chair to complete this test; or due to recent surgery, pain, physical limitations or use of assistive devices, and the need to adapt test administration when caregivers could not lift their right leg to the12–16 inch height stipulated by Step Test guidelines [29].

#### Intervention implementation

Caregivers could participate in 20 telephone intervention calls over 12 months for a maximum of 375 min. EPAI caregivers participated in an average of 14 telephone calls. The EPAI had significantly fewer telephone calls (i.e., 14 out of 20=70% averaging 25 min/call); while CSBI caregivers participated in 18 telephone calls averaging 21 min/call (*p=0.01*). However, total telephone minutes did not significantly differ by group (*p*=0.07; Table 2).

## Discussion

### Study hypotheses

Given the prevalence of ADRD, the need for family care, and the toll that caregiving takes on family members’ mental and physical health (2), this study addressed a major public health problem-increasing physical activity of sedentary caregivers. Findings supported both study hypotheses. First, a significantly higher percentage of EPAI than CSBI caregivers, demonstrated adherence to MVPA. A major contribution of this study was that the EPAI was more effective in increasing MVPA than the control group- 63% versus 37%; which places EPAI participants amongst the 60–63% of older adults in the United States, age 60–69 years, who met recommendations for increasing MVPA. Meeting this recommendation has potential positive implications for caregiver health, as this level of physical activity has been associated with reduced risk of chronic disease, premature mortality, and improved functional abilities [46]. These findings also highlighted the ability of caregivers to balance their own health-related and functional needs alongside their care recipient needs [19,47,48].

The second hypothesis, improving caregiver physical function, was supported by the 2 min Step Test but not the 30 s Chair Stand. These findings highlighted the importance of family caregivers needing to maintain physical function, so as to safely implement care for their impaired family member, as well as maintaining their own health and safety. However qualitative findings, which suggested that nearly one-half of the caregivers had difficulty completing the 2 min Step Test, due either to their own high blood pressure or cardiovascular limitations, highlights the potentially dangerous situations that selected caregivers and care recipients face. This level of aerobic endurance is very important for caregivers’ physical activities, including stair climbing, walking, transferring their impaired relative from bed to chair or even turning and moving a bed-bound relative [29]. Of note, is that the overall average of 60–62 steps reported at baseline and 12 months for the Step Test, is more similar to persons who would be classified as inactive older adults, ranging from 85–89 years, suggesting that numerous caregivers were considerably less active and more impaired than other persons their own age. This same line of reasoning holds true for the Chair Stand test where TRAC caregivers’ mean number of Chair Stands was slightly lower than what is reported for 80–89 year olds [29].

Other background health-related information noted that caregivers’ average BMI and weight classified them as being within the range of 43–45% of men and women, respectively, between 65–74 years, who would be classified as being obese [49]. Obesity creates greater risk for caregiver physical strain, and also places greater demands upon caregivers when implementing care-related activities such as ambulating and/or transferring care recipients from bed to chair, or moving them in bed. However, similar to our findings, an earlier study found that physical activity measures were generally not correlated with health measures [49].

The TRAC study was designed so that caregivers in both interventions had access to family caregiving content, as we hypothesized that family caregivers had different needs and responsibilities than other older adults who engage in physical activity, who are not family caregivers. The EPAI was designed to focus on the goal of increasing MVPA, but to also provide participants access to needed caregiving information and support, that might interfere with this goal. The CSBI group was designed to provide caregivers basic information and support concerning caregiving, and also to control for level of intervention dose/time. The CSBI was based on existing caregiver psychoeducational interventions [6,50–53].

Three caregiver variables significantly differed between the interventions from baseline to 12 months, with CSBI caregivers reporting *increased hours of caregiving, more use of formal resources* and participating in significantly *more telephone sessions*. We hypothesize that the CSBI may have increased their hours of caregiving and formal service use because they were implementing caregiving skills that were addressed during the CSBI [21,41,53]. Findings that no other stressor or resource variables differed by intervention over time, suggested that EPAI caregivers benefited from receiving caregiver related information and support, but that EPAI caregiving content did not exceed that provided by the CSBI.

That the EPAI received significantly fewer telephone intervention implementation calls may be explained by how each intervention was structured, where the EPAI was structured around setting physical activity goals, reviewing progress toward meeting activity goals, and identifying how goals needed to be adapted before the next intervention session [26,54–56]; while the CSBI was structured around supporting caregivers regarding caregiving skill building and emotional support, and attaining goals that were most relevant for care recipient and caregiver-related issues [21]. It is possible that the CSBI, as a supportive intervention, took more caregiver and interventionist’s time. However, data suggested that the CSBI 29 min of extra intervention time, did not result in significantly different outcomes between the two interventions concerning caregiving stressors, resources or background health information; and data supported that the more directive EPAI was successful in increasing caregiver MVPA.

### Limitations

Study implementation was complex, considering the sample size and number of contacts needed to recruit a sufficiently powered sample. Restricted selection criteria presented potential barriers, as did caregiver personal physical activity preferences and limitations. To address potential attrition bias, we closely monitored the study process and reasons for attrition; used analytic approaches to understand which caregivers dropped out and why, and maximized use of available data. Considerable EPAI attrition occurred within the first three study months (22%), similar to other studies of older adults, that reported first-year attrition rates of 22% to 76%, with greatest dropouts occurring during the first three study months [57]. An unexpected finding was that White caregivers were more likely to drop out than Blacks and other minorities. This difference may have been due to healthcare disparities, in which minorities often have less access to facilities and services; and minorities may have experienced the study as a clinical resource, not otherwise available to them [58].

Study personnel used approaches commonly identified as beneficial when working with hard-to-reach populations [22]. Recruitment and intervention staff were experienced in establishing trust, confidence and rapport with caregivers; as well as using culturally appropriate approaches in working with caregivers [59]. Staff adapted study procedures to older adult and caregiver needs, such as conducting interviews in caregiver homes, by telephone, or met with caregivers in other places, such as public libraries that had private space for confidential interviews. Some older adults view multiple data collection follow-ups as burdensome. Research staff worked with caregivers to divide interviews into shorter sessions; or agreed to shorten the interview by collecting only primary outcome data [60]. Other researchers who conduct longitudinal studies have found that healthier and less disabled persons are more likely to volunteer to participate in these studies; that decreased attendance after baseline is common in studies with longitudinal follow-up; and is often related to age, health and physical function [60]. Longitudinal studies with older adults can be difficult, expensive and time-consuming [61] and potential retention bias may affect magnitude of changes seen in study outcomes, such as increased MVPA and physical function, as well as body composition and strength [60]. A second study limitation included use of a self-report measure of physical activity.

Upon recruitment into the study, caregivers reported *not* engaging in ≥ 60 min of physical activity/week, but when *reporting* baseline physical activity via CHAMPS, they reported somewhat more physical activity minutes/week. EPAI caregiver weekly logs and pedometer steps helped to confirm that over time, caregivers were increasing their physical activity; the CHAMPS measure was sensitive to change and detected increased EPAI levels of total MVPA [25]. It is also possible that caregivers initially underestimated their physical activity involvement, but when they were assessed using the CHAMPS, they became more aware of moderate/vigorous types of physical activities in which they were engaging. An earlier pilot study also supported validity between objective and self-report physical activity data [26].

Recommendations for the future studies to improve family caregiver physical activity research should emphasize understanding inactivity and the synergy between physical activity and other health behaviors [62]. A large European cohort study of older adults noted that greatest reductions in mortality risk were observed in the two *lowest* activity groups across levels of general and abdominal adiposity. Researchers suggested that: (1)”efforts to encourage even small increases in activity in inactive individuals may be beneficial to public health” [63, p.620]; (2) research also suggests that including self-report and objective measures of physical function assists in characterizing activity patterns and physical functional abilities, and increases understanding of how physical activity and physical function are interrelated; and types of physical activities that may assist older adults in making small physical activity changes [29]; (3) further refining program-based components including recruitment and intervention procedures to identify and support caregivers who are most likely to participate, increase physical activity, and remain in, and benefit from a physical activity study; (4) tailoring physical interventions to individual caregiver needs and understanding the minimum level of physical activity needed to produce health effects; (5) recruiting more multicultural family caregivers, considering their needs and specifically tailoring interventions to meet these needs [64], and (6) considering technologically-based methods for delivering physical activity interventions to this time-pressured, harder-to-reach population [62,65].

## Conclusion

A major contribution of this study was that it used a combination of self-report physical activity and objective physical function outcome data, which may help caregivers to more readily experience how increased physical activity may improve physical function. Limitations included that some caregivers had greater physical function impairment than originally expected. These limitations may have influenced differential attrition between the EPAI and CSBI and may have affected the magnitude of changes seen in MVPA [60]. Recommendations for future caregiver physical activity studies include: (1) Further develop interdisciplinary research teams prepared to address the combination of caregiver education and support, as well as improvement of physical activity and physical function, as a broader approach to maintaining caregiver health; (2) Design studies that tailor physical activity interventions to caregiver abilities and functional limitations, and further address underlying reasons for caregiver attrition; (3) Expand use of internet-based modalities to increase reach and flexibility of caregiver physical activity studies [56,65]; and (4) Translate effective physical activity interventions for greater dissemination to family caregivers [5].

## Figures and Tables

**Table 1:** Caregiver and Care Recipient Baseline Characteristics; Caregiver Stressors, Resources and Background Health by Treatment Condition: Baseline and 12 Months.

Variable	EPAI	CSBI	*p*
	n=106	n=105	
	M (SD)	M (SD)	
**CG Baseline Characteristics**			
Age in years	61 (12)	62 (13)	0.43
**CR Baseline Characteristics**			
Age in years	79 (10) 59	80 (9) 68	0.69
Mini Mental State Exam	16 (8)	15 (8)	0.22
**CG Stressors**			
PADL/IADL			
Baseline	2.6 ± 1	2.5 ± 1	
12 months (n=126)	2.7 ± 1^a^	2.7 ± 1^b^	0.53
Behavioral Symptoms			
Baseline	7.8 ± 2.7	7.4 ± 3	
12 months (n=126)	7.5 ± 2.8^a^	7.3 ± 3 b	0.38
Hours of Caregiving			
Baseline	33 ± 21	35 ± 26	
12 months (n=126)	32 + 2^a^	42 ± 81^b^	<0.001
**CG Resources**			
Total Formal Services			
Baseline	1.7 ± 1	1.6 ± 1	
12 months (n=126)	1.6 ± 1^a^	1.8 ± 1^b^	0.02
Perceived Social Support			
Baseline	11 ± 4	12 ± 3	
12 months (n=126)	11 ± 4^a^	12 ± 4 b	0.78
**CG Background Health**			
Chronic Conditions			
Baseline (n=210)	2.1 ± 1.7	2.1 ± 1.3	
12 months (n=114)	2.2 ± 1.6	2.3 ± 1.3	0.3
Total Medications			
Baseline (n=211)	5.5 ± 4.0	5.6 ± 4.0	
12 months (n=126)	2.9 ± 3.4 a	3.7 ± 4.4 b	0.06
Body Mass index			
Baseline (n=211)	28.6 ± 6	29.7 ± 7	
12 months (n=114)	29.4 ± 4	29.5 ± 8	0.34
Weight in Pounds			
Baseline (n=211)	173.6 ± 40.8	180.3 ± 45	0.66
12 months (n=121)	177.3 ± 42.5	177.8 ± 46	
Systolic blood pressure			
Baseline (n=211)	125.1 ± 16.2	127.2 ± 17.1	0.16
12 months (n=117)	123.1 ± 15.1	123.8 ± 17.6	
Diastolic blood pressure			
Baseline (n=211)	73.5 ± 10.6	74.8 ± 10.0	
12 months (n=117)	72.5 ± 11.0	72.6 ± 11.6	0.18

Note. CG=Caregiver; CR=Care Recipient; CSBI=Caregiver Skill Building Intervention; EPAI=Enhancing Physical Activity Intervention; *M*=Mean; n=number; PADL/IADL=Personal Activities of Daily Living/Instrumental Activities of Daily Living; *SD*=Standard Deviation; Sample size for

a EPAI: 12 month data=53

b CSBI: 12 month data=73

**Table 2:** Caregiver Primary and Secondary Outcomes, and Intervention Implementation by Treatment Group at Baseline and 12-Months.

Caregiver Variables	EPAI	CSBI	*p*
	*n*=106	*n*=105	
	*M SD*	*M SD*	
**Primary Outcome**			
Total MVPA			
Baseline (n=211)	62 ± 119	79 ± 111	0.09
12 months (n=126)	133 ± 167	59 ± 88	≤ 0.001
**Secondary Outcome**			
Physical Function			
2 Minute Step Test			
Baseline (n=92)	59.2 ± 25.9	60.7 ± 25.1	
12 months (n=57)	66.8 ± 24.5	56.8 ± 30.3	0.4
30-Second Chair Stand			
Baseline (n=207)	10.0 ± 3.0	10.2 ± 3.4	
12 months (n=116)	10.5 ± 3.9	10.6 ± 3.3	0.24
**Intervention Implementation** (Total=20)			
Total sessions attended	14 ± 6	18 ± 5	0.01
Total intervention time (min)	354 ± 166	383 ± 123	0.07

**Table 3:** GEE Models for EPAI Caregiver Physical Activity and Physical Function by Time, Main Effect and Group-by-Time Interaction: Baseline to 12 Months.

	CHAMPS Physical Activity	SFT Physical Function

Outcome	Log Transformed MVPA Est (stnd error)	2-Minute Step Test Est (stnd error)

# Subjects	155	134
Total Observations	428	347
Baseline	4.34***(0.15)	61.60***(3.20)
EPAI	−0.05(03.28)	−0.64 (4.89)
Month 6	−0.03 (0.18)	−5.84* (2.57)
Month 12	−0.25 (0.16)	−7.61** (2.71)
EPAI_Month 6	0.57* (0.27)	9.72* (3.91)
EPAI_Month 12	0.77** (0.27)	12.72** (4.15)

*Note*.CHAMPS=Community Healthy Activities Model Program for Seniors; EPAI=Enhancing Physical Activity Intervention; est=estimate; GEE=Generalized Estimating Equation Models; MVPA=Moderate to Vigorous Physical Activity; SFT=Senior Fitness Test; std.=standard error

* p<0.05,

** p<0.01,

*** p<0.001.

## Abbreviations

ADRD: Alzheimer’s Disease and Related Dementias
CG: Caregiver
CR: Care Recipient
CSBI: Caregiver Skill Building Intervention
EPAI: Enhancing Physical Activity Intervention
METS: Metabolic Equivalents
MMSE: Mini Mental State Examination
MVPA: Moderate to Vigorous Physical Activity
PADL/IADL: Personal Activities of Daily Living/Instrumental Activities of Daily Living
RA: Research Assistant
RADC: Rush Alzheimer’s Disease Center
RCT: Randomized Controlled Trial
SFT: Senior Fitness Test
TRAC: Telephone Resources and Assistance for Caregivers
